# Interaction between DNA Polymerase β and BRCA1

**DOI:** 10.1371/journal.pone.0066801

**Published:** 2013-06-27

**Authors:** Aya Masaoka, Natalie R. Gassman, Julie K. Horton, Padmini S. Kedar, Kristine L. Witt, Cheryl A. Hobbs, Grace E. Kissling, Keizo Tano, Kenjiro Asagoshi, Samuel H. Wilson

**Affiliations:** 1 Laboratory of Structural Biology, NIEHS, National Institutes of Health, North Carolina, United States of America; 2 National Toxicology Program, NIEHS, National Institutes of Health, North Carolina, United States of America; 3 Biostatistics Branch, NIEHS, National Institutes of Health, North Carolina, United States of America; 4 Integrated Laboratory Systems, Inc., North Carolina, United States of America; 5 Department of Radiation Life Science and Radiation Medical Science, Kyoto University Research Reactor Institute, Kumatori, Japan; University of Texas Health Science Center at San Antonio, United States of America

## Abstract

The breast cancer 1 (BRCA1) protein is a tumor suppressor playing roles in DNA repair and cell cycle regulation. Studies of DNA repair functions of BRCA1 have focused on double-strand break (DSB) repair pathways and have recently included base excision repair (BER). However, the function of BRCA1 in BER is not well defined. Here, we examined a BRCA1 role in BER, first in relation to alkylating agent (MMS) treatment of cells and the BER enzyme DNA polymerase β (pol β). MMS treatment of BRCA1 negative human ovarian and chicken DT40 cells revealed hypersensitivity, and the combined gene deletion of BRCA1 and pol β in DT40 cells was consistent with these factors acting in the same repair pathway, possibly BER. Using cell extracts and purified proteins, BRCA1 and pol β were found to interact in immunoprecipitation assays, yet *in vivo* and *in vitro* assays for a BER role of BRCA1 were negative. An alternate approach with the human cells of immunofluorescence imaging and laser-induced DNA damage revealed negligible BRCA1 recruitment during the first 60 s after irradiation, the period typical of recruitment of pol β and other BER factors. Instead, 15 min after irradiation, BRCA1 recruitment was strong and there was γ-H2AX co-localization, consistent with DSBs and repair. The rapid recruitment of pol β was similar in BRCA1 positive and negative cells. However, a fraction of pol β initially recruited remained associated with damage sites much longer in BRCA1 positive than negative cells. Interestingly, pol β expression was required for BRCA1 recruitment, suggesting a partnership between these repair factors in DSB repair.

## Introduction

It is well known that inactivation of the product of the breast cancer 1 (BRCA1) gene is linked to susceptibility to early-onset breast and ovarian cancer [1], [2], and inactivating mutations in the gene confer high lifetime risk of cancer. The human BRCA1 gene is organized into 24 exons and encodes a 220-kDa protein. The BRCA1 protein is generally considered to be both a tumor suppressor and a DNA repair factor involved in multiple DNA repair and genome stability processes [3], [4]. BRCA1 is found in the nucleus and has a single conserved amino-terminal RING domain, and 2 tandem BRCT domains at the carboxy-terminus. The RING domain of BRCA1 is required for its interaction with BRCA1-associated RING domain protein1 (BARD1) [5]. The BRCT domains of BRCA1 are responsible for phosphorylation-dependent localization, and these domains are thought to regulate multiple pathways, including those responsible for tumor suppression.

Much of the DNA repair research associated with BRCA1 [6], [7] has focused on homology-directed DNA double-strand break (DSB) repair by homologous recombination (HR) as well as non-homologous end joining. However, BRCA1 is also involved in nucleotide excision repair (NER) [8], and recently Alli et al. demonstrated a role of BRCA1 in the repair pathway termed base excision repair (BER) [9]. Regarding the role of BRCA1 in HR, DSBs are recognized by the MRN complex and repair is initiated by resection of one of the DNA strands by MRE11, a component of this complex. The resulting single-stranded DNA is coated by replication protein A (RPA), and RAD51 then replaces RPA and facilitates strand invasion at homologous DNA regions, eventually forming a Holliday junction. This junction is resolved and DNA ends are ligated to complete HR repair. BRCA1 facilitates the HR process through signaling of cell cycle arrest, DSB binding and interaction with the MRN complex and RAD51. However, BRCA1 is not absolutely required since loss of 53BP1 specifically in BRCA1-mutant cells abrogates the ATM checkpoint response and partially restores the HR pathway [10], [11]. DNA binding properties of BRCA1 could also facilitate its roles in the NER [8] and BER pathways, but these roles are not yet defined.

The term BER applies to several base lesion DNA repair sub-pathways related to the type of lesion being repaired, the excision patch size, the excision gap trimming activities, and the DNA glycosylase involved in initiating the pathway. The enzymatic requirements for the various sub-pathways are quite distinct. In the case of the oxidative stress-induced base lesion, 8-oxoguanine, BER is initiated by the lesion specific glycosylase, 8-oxoguanine DNA glycosylase (OGG1). This repair sub-pathway was found to be deficient in BRCA1-deficient cells and these cells were strongly hypersensitive to hydrogen peroxide treatment, that leads to oxidized DNA [9]. The mechanistic basis for the BRCA1 requirement is unknown, but the BRCA1-deficient cells accumulated higher levels of strand break intermediates than cells expressing wild-type BRCA1 thus confirming the existence of a DNA repair deficiency. Conversely, BRCA1 is reported to stimulate BER of oxidative DNA damage by enhancing the activity of BER enzymes including OGG1, the DNA glycosylase NTH1 and AP endonuclease 1 [12].

In the present study, we further examined BER sub-pathways for dependence on BRCA1. Exposure of cells to methyl methanesulfonate (MMS) triggers methylation of DNA bases and oxidative stress-induced base damage, among other responses [13], [14], [15]. In the case of methylated purine bases, the BER intermediate formed by the action of the monofunctional alkyladenine DNA glycosylase (AAG) is different from the intermediate formed by bifunctional OGG1. With methylated base removal, AAG produces an abasic site in double-stranded DNA. This repair intermediate is incised adjacent to the abasic site by AP endonuclease 1, leaving a 1-nucleotide gap with a 5′-deoxyribose phosphate (5′-dRP) group at the 5′-margin. This intermediate is processed by the 5′-dRP lyase and 1-nucleotide gap-filling activities of pol β. In contrast, the OGG1-generated BER intermediate has a blocked 3′-margin in the 1-nucleotide gap that must be tailored by other enzymes before the gap can be filled by pol β.

Thus, the BER sub-pathways for methylated and oxidized purine bases are different and may exhibit different roles for BRCA1. However, a common feature of these BER sub-pathways is that pol β is the primary gap-filling polymerase and could potentially interact with BRCA1 during repair of both types of lesions. Here we further examine a role for BRCA1 in BER and report a partnership between BRCA1 and pol β in protecting cells against the MMS-induced cytotoxicity. The mechanism of the synergistic protective effect involving these repair factors, BRCA1 and pol β, was further explored.

## Materials and Methods

### Cell lines and culture

BRCA1 functionally-deficient human ovarian cancer cells (UWB1.289) and cells complemented with wild-type human BRCA1 (UWB1.289+BRCA1) were from the American Type Culture Collection (Manassas, VA). These cell lines were maintained in 50% RPMI 1640 (Invitrogen, Carlsbad, CA) + 50% MEGM (Lonza Group Ltd., Walkersville, MD) supplemented with 3% fetal bovine serum (HyClone, Logan, UT) and 2 mM L-glutamine (Invitrogen) in a 5% CO_2_ incubator at 37°C. The UWB1.289+BRCA1 cell line was maintained in the same medium with 200 μg/mL G418 (Invitrogen). Mycoplasma testing was performed using a MycoAlert® Mycoplasma detection kit (Lonza, Rockland, ME) and all cells were found to be free of mycoplasma contamination.

The wild-type chicken lymphoma B cell line DT40 was a gift from Dr. S. Takeda and has been maintained in the Takeda laboratory since 1991 [16]. These cells and isogenic DT40-derived cell lines were maintained in RPMI 1640 supplemented with 10% fetal bovine serum, 1% chicken serum (Invitrogen), 50 μM mercaptoethanol (Sigma-Aldrich, St. Louis, MO) and 2 mM L-glutamine in 5% CO_2_ incubator at 39.5°C. Variants were engineered as described previously [16], [17] using gene targeting constructs for *BRCA1* [18], [19] and *Pol β* [20]. The double knock out *BRCA1^−/−^/Pol β^−/−^* cell line was generated by targeted disruption of the pol β gene in the *BRCA1^−/−^* background using pPol β-Bsr then pPol β-His constructs [20] (Figure S1B). Targeted integration of these constructs was expected to delete amino acids 77–213 of pol β, spanning the 8 kDa dRP lyase domain and the N-terminal portion of the 31 kDa polymerase domain [20]. The absence of these loci in the knockout cell lines was confirmed by Southern blot analysis (Figure S1A).

### Preparation and transfection of the uracil-DNA plasmid for the *in vivo* BER assay

The plasmid containing uracil was prepared as described previously [21]. AM1 plasmid was improved to prevent any residual background luciferase levels from un-replaced original plasmid in the transfected DNA. The AM1-stop plasmid was constructed from the AM1 with a stop codon at the BsaXI replaceable region. An oligonucleotide containing uracil at a specific site was inserted into the BsaXI site of the AM1-stop plasmid to produce AM1-U. After ligation, the product was electrophoresed in a 1% agarose gel and visualized using a BlueView Transilluminator. Recircularized DNA plasmids were isolated and extracted using a gel extraction kit (Qiagen) and suspended in TE buffer.

The UWB1.289 and UWB1.289+BRCA1 cell lines were plated (1×10^5^ cells/well) in a 96-well flat clear bottom polystyrene tissue culture-treated plate (Corning, Lowell, MA) and incubated overnight. The next day, cells were washed and the medium was replaced with Opti-MEM I Reduced Serum Medium without phenol red (Invitrogen). Uracil-DNA plasmid (200 ng) and an equal amount of internal control plasmid (pGL4.75) per well were mixed with PLUSReagent (Invitrogen) and Lipofectamine LTX Reagent (Invitrogen) in the Opti-MEM I Reduced Serum Medium without phenol red, and the mixture was used for transfection. Transfections were performed on ice for 3 h, followed by medium change to DMEM without phenol red containing 10% fetal bovine serum, 4 mM GlutaMAX-I, 25 mM HEPES and 0.2 mM D-luciferin potassium salt (MP Biomedicals, Solon, OH). Data were taken after 18 h of repair following continuous measurement of luciferase activity at 37°C using a Tropix TR717 microplate luminometer (Applied Biosystems Inc., Foster City, CA). Each well was washed with phosphate buffered saline (PBS). In some cases, cell lysates were prepared and activities were assayed using the Dual-Glo Luciferase assay system (Promega).

### Cytotoxicity assay

Cytotoxicity was determined by growth inhibition assays as described previously [22]. UWB1.289 and UWB1.289+BRCA1 cell lines were seeded at a density of 40,000 cells/well in 6-well plates 24 h before treatment. The following day, cells were exposed to potassium bromate (KBrO_3_), hydrogen peroxide (H_2_O_2_) or methyl methanesulfonate (MMS) (Sigma-Aldrich) for 1 h at 37°C in growth medium. Cells were washed with Hanks' balanced salt solution (HyClone) and fresh medium was added. Pamoic acid (PA; 300 µM) (Sigma-Aldrich) was prepared in the growth medium and cells were pre-exposed for 7 h before MMS treatment. PA remained during and after MMS treatment, and the medium was replaced after a total of 24 h incubation with PA. The cells then were incubated for approximately 7 days in a 5% CO_2_ incubator at 37°C until untreated control cells were approximately 80% confluent. Cells (triplicate wells for each drug concentration) were counted by a cell lysis procedure, and results were expressed as the number of cells in drug-treated wells relative to untreated control cells (% control growth) [23].

For DT40 cells, colony formation was assayed in medium containing methylcellulose as previously described [24]. To determine the sensitivity to MMS, DT40 cells were plated in triplicate in MMS-containing medium in 60-mm plates and were incubated at 39.5°C for 6 to 7 days. Colonies were counted, and % survival was determined relative to the number of colonies obtained from non-treated cells.

### *In vitro* cell extract-based BER assay

Whole cell extracts were prepared as described [25]. Briefly, cells were grown to near-confluency, washed twice with cold PBS, detached by scraping, collected by centrifugation, and suspended in Buffer I (10 mM Tris-HCl, pH 7.8, 200 mM KCl, and protease inhibitor cocktail) (Roche Molecular Diagnostics, Pleasanton, CA). An equal volume of Buffer II (10 mM Tris-HCl, pH 7.8, 200 mM KCl, 2 mM EDTA, 40% glycerol, 0.2% Nonidet P-40, 2 mM dithiothreitol and protease inhibitor cocktail) was added. The cell suspension was mixed in the rocking platform for 1 h at 4°C, and the resulting extract was clarified by centrifugation at 14,000 rpm at 4°C. The protein concentration was determined by Bio-Rad protein assay (Bio-Rad Laboratories, Hercules, CA) using bovine serum albumin (BSA) as standard.

The *in vitro* cell extract-based BER assay was performed with a 35-bp oligonucleotide DNA substrate containing uracil, 5′-GCCCTGCAGGTCGA**U**TCTAGAGGATCCCCGGGTAC, annealed with the complementary strand, 5′-GTACCCGGGGATCCTCTAGAGTCGACCTGCAGGGC. The duplex oligonucleotide substrate at a final concentration of 200 nM was incubated with 10 μg of whole cell extract prepared from UWB1.289 and UWB1.289+BRCA1 cells. In addition, the reaction mixture contained 50 mM Tris-HCl, pH 7.5, 5 mM MgCl_2_, 20 mM NaCl, 0.5 mM DTT, and 4 mM ATP. The incubation was conducted in the presence of 20 μM each dATP, dGTP, and dTTP, and 2.3 μM [α-^32^P] dCTP (3,000 Ci/mmol, GE Healthcare, Piscataway, NJ) at 37°C for 2, 5, and 10 min. The reaction products were analyzed by 15% polyacrylamide denaturing gel electrophoresis.

### DNA damage and repair evaluated by the comet assay

The comet assay was used to measure MMS-induced single strand breaks at 0 and 60 min after exposure as an indicator of the status of BER in wild-type and mutant DT40 cell lines lacking a functional BRCA1 gene. On the day of experimentation, viability was confirmed at >90% using trypan blue staining, cells were diluted to a concentration of 0.5-1×10^6^ cells/mL then layered onto prepared slides as follows: Microscope slides were dipped in 1% normal melting point agarose in Ca^+2^ and Mg^+2^ free PBS (Sigma-Aldrich) and stored in a desiccator at room temperature for up to 24 h prior to use. An aliquot of 1% NuSieve^®^ GTG^®^ low melting point (LMP) agarose (Lonza, Rockland ME) was allowed to equilibrate to 37°C in a water bath. A 1∶10 ratio of cells to LMP agarose was gently mixed by pipetting (250 µL of cells to 2.5 mL of LMP) and 100 µL of this cell suspension was layered onto a pre-coated slide, immediately coverslipped, and chilled at 4°C. After the agarose solidified, the coverslip was removed, a second layer of 100 µL 1% LMP was layered onto the slide, and the slide was immediately coverslipped. Slides were chilled for approximately 10 min at 4°C.

MMS concentration and repair time points were selected on the basis of results from previous studies [22]. Slides were arranged in a horizontal slide holder and one set of slides from each cell line was exposed to 20 mM cold MMS prepared in complete medium in a refrigerated glass staining dish for 20 min. Following MMS exposure, treated slides were quickly washed once in cold PBS for a few seconds and the cells were allowed to undergo repair for 0 or 60 min in pre-warmed complete growth medium in a 39.5°C incubator. At either 0 or 60 min, slides for each cell line per time point were immediately put into cold lysing solution (2.5 M NaCl, 100 mM Na_2_EDTA, 10 mM Tris, pH 10.0, with 10% dimethylsulfoxide and 1% Triton X-100 added fresh). A second set of slides for each cell line was incubated in refrigerated complete medium for 20 min, and then handled in the same fashion as the treated cells. These untreated slides were lysed in separate coplin jars (to avoid any carryover contamination with MMS) to serve as concurrent negative controls for MMS-induced damage. The slides remained in lysing solution overnight at 4°C. Following lysis, slides were washed for 5 min in 0.4 M Tris-HCl, pH 7.5 before placing into cold electrophoresis buffer (0.3 M NaOH, 1 mM Na_2_EDTA, pH>13) in an electrophoresis chamber for 20 min at <10°C to allow the DNA to unwind. Slides were then electrophoresed at 25 V (∼300 mA) for 40 min at <10°C, and then washed 3 times (5 min each) with 0.4 M Tris-HCl, pH 7.5, and fixed in cold ethanol (Pharmco-AAPER, Shelbyville, KY) for at least 30 min. NaCl, Na_2_EDTA, Triton X-100 and Trizma base were purchased from Sigma-Aldrich; NaOH was purchased from RICCA Chemicals (Arlington, TX) and dimethylsulfoxide from Fisher Scientific (Pittsburgh, PA). Slides were air-dried and stored in a desiccator with <60% humidity until analysis.

All slides were coded prior to evaluation. After staining slides with SYBR^TM^ Gold (Molecular Probes, Invitrogen), 100 cells were scored per slide using Comet Assay IV image analysis software Version 4.11 (Perceptive Instruments, Ltd., Suffolk, UK). The extent of DNA damage was measured as the percentage of migrated DNA (% Tail DNA).

Prior to analyzing the data, the mean % Tail DNA and the standard deviation (SD) of the % Tail DNA for each slide of the MMS-exposed cells was expressed as a percent of the untreated % Tail DNA of the same cell type at the same time point. In particular, “Adjusted Mean” and “Adjusted SD” values were calculated for the MMS-exposed % Tail DNA of each slide:



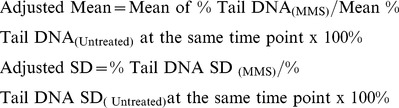



Two-way analysis of variance (ANOVA) on the Adjusted Means of the MMS-exposed cells, with cell line and time as the two factors and including a cell line × time interaction term was used to investigate whether the effect of cell line was consistent over time. To compensate for unequal variances, the data were weighted by the inverse of the Adjusted SD (i.e., 1/Adjusted SD) in the ANOVAs. Within each cell line, Dunnett's test was used to determine if the Adjusted Mean differed from the mean at the 0 time point.

### Immunofluorescence

The detailed method is as described previously [26]. Briefly, UWB1.289 or UWB1.289+BRCA1 cells (2×10^5^) were seeded onto 35-mm glass bottomed petri dishes (MatTek, Ashland, MA) and incubated in cell culture medium containing 10 mM BrdU (Sigma-Aldrich) for 24 h. After 24 h, medium was exchanged to complete medium without BrdU. Samples were then imaged using a 40× C-Apochromat (numerical aperture 1.2) water immersion objective coupled to a Zeiss LSM510 META confocal microscope (Carl Zeiss MicroImaging). DNA damage was introduced by UV laser micro-irradiation at 364 nm (Coherent Enterprise II) with intensities equivalent to 0.44 mJ. After micro-irradiation, cells were either immediately fixed in 4% paraformaldehyde or allowed to recover in a 37°C incubator for the times noted. After fixation, cells were permeabilized with 1% SDS in PBS for 10 min, washed 5 times in PBS, then further permeabilized and blocked with PBS + 1% BSA for 30 min. Cells were then incubated with a 1∶200 dilution of anti-pol β antibody (ab26343, Abcam Cambridge, MA) and 1:100 dilution of anti-BRCA1 antibody (OP92, EMD Millipore, Darmstadt, Germany) for 1 h. Cells were washed 3 times in PBS, then incubated in 1:2500 dilution of Alexa 488 conjugated anti-mouse and Alexa 546 conjugated anti-rabbit (Invitrogen) antibody for 1 h. Finally, fluorescence images were acquired with the 40× water immersion objective on the LSM510. Recruitment of BRCA1 or pol β to the site of DNA damage was measured using IMAGEJ. The mean intensity of the irradiation line was determined after subtraction of the background intensity in the irradiated cell.

### Immunoblotting of BRCA1 and other BER proteins

Immunoblotting analysis was carried out as described [27]. Cell extracts (40 µg) were loaded onto NuPAGE 4–12% Bis-Tris gels (Invitrogen), and proteins were separated by electrophoresis and transferred onto a nitrocellulose membrane. The membrane was incubated with 5% nonfat dry milk in Tris-buffered saline containing 0.1% (v/v) Tween 20 (TBST) to reduce non-specific binding. The membrane was probed with antibodies as follows: BRCA1 (OP92, EMD Millipore), pol β (18S) [28], FEN1 (ab17993, Abcam), PARP-1 (51-6639GR, BD Pharmingen, San Diego, CA), XRCC1 (33-2-5, Thermo Fisher Scientific, Kalamazoo, MI), ligase I (GTX70141, GeneTex Inc. Irvine, CA), ligase III (GTX70143, GeneTex), and GAPDH (G3PDH11-M, Alpha Diagnostic, San Antonio, TX) at an appropriate dilution in nonfat dry milk in TBST for 2 h at room temperature. After washing with TBST, the membrane was incubated with secondary antibody, either goat anti-mouse or goat ant-rabbit IgG (H+L)-horseradish peroxidase (HRP) conjugate (Bio-Rad Laboratories) for 1 h at room temperature. HRP activity was detected by enhanced chemiluminescence using SuperSignal West Pico Chemiluminescent substrate (Pierce Biotechnology Inc., Rockford, IL). The antibodies on the membrane were stripped by incubation with Restore Western Blot Stripping Buffer (Pierce Biotechnology Inc.) for 30 min at room temperature. After washing with TBST, the membrane was subjected to immunodetection with other antibodies.

### Co-immunoprecipitation of BRCA1 and pol β

The human BRCA1 cell lines were cultured in 150-mm dishes until near confluence, and cell lysates were prepared in a lysis buffer (50 mM Tris-HCl, pH 7.5, 150 mM NaCl, 25 mM NaF, 0.1 mM sodium orthovanadate, 0.2% Triton X-100, 0.3% Nonidet P-40 with protease inhibitor cocktail) as described previously [29]. For co-immunoprecipitations, anti-BRCA1 polyclonal antibody (GTX29141, GeneTex) or anti-pol β polyclonal antibody [28] was added to the cell lysate (1 mg protein), and the mixture was incubated with rotation for 4 h at 4°C. The immunocomplex was adsorbed onto protein A-sepharose (GE Healthcare, Piscataway, NJ) and protein G-agarose (Roche Molecular Biochemicals) beads by incubating the mixture overnight at 4°C. The beads were washed 4 times with lysis buffer containing protease inhibitors. Beads were resuspended in SDS-PAGE sample-buffer, heated for 5 min at 95°C, and the soluble proteins were separated in a NUPAGE 4–12% Bis-Tris gel (Invitrogen). Proteins were then transferred onto a nitrocellulose membrane and were analyzed as described above. Co-immunoprecipitation of purified BRCA-1 protein (ab82204, Abcam) or purified pol β [30] was carried out in a binding buffer (25 mM Tris-HCl, pH 8.0, 10% glycerol, 100 mM NaCl, 0.1% NP-40) containing protease inhibitors. A 1.5 µM equal molar protein mixture of BRCA1 and pol β was combined with either anti-BRCA1 polyclonal antibody (sc-641 D-20, Santa Cruz Biotechnology, Inc., Santa Cruz, CA) or anti-pol β polyclonal antibody [28] in a final volume of 50 µL. The mixtures were treated and analyzed as described for the co-immunoprecipitations of cell extracts, and the complexes were adsorbed onto protein A-sepharose and protein G-agarose beads in a final volume of 500 µL of binding buffer. In control experiments, the antibody for immunoprecipitation was substituted with rabbit pre-immune IgG (Sigma-Aldrich). Cell extracts (50 µg) and purified proteins (100 ng each), without immunoprecipitation, were used as marker for pol β and BRCA1, as indicated.

### Pol β gene knockdown

Pol β shRNA was purchased from Addgene (Cambridge, MA) and used for knockdown of pol β expression as described previously [25]. Briefly, BRCA1 cells were transfected with control plasmid or pol β-specific shRNA using Lipofectamine LTX Reagent (Invitrogen). Knockdown clones were selected with 1 µg/mL puromycin, and the reduced expression of pol β was confirmed by immunoblotting analysis.

## Results

We examine a role for BRCA1 in cellular protection against alkylating agent MMS-induced stress as a function of pol β expression. The MMS stress response is known to involve multiple pathways including the BER pathway. A variety of tools for study of BER, and a putative BRCA1 and pol β interaction were used. These included measurement of MMS-induced cytotoxicity in cells with pol β and BRCA1 gene deletions, assays for the BER capacity of cells, analysis of pol β and BRCA1 protein-protein interaction, and imaging studies of recruitment of these factors after laser-delivered focal UV irradiation damage in cells. A total of 5 cell lines were chosen for study: a) a BRCA1 mutant human ovarian cancer cell line termed UWB1.289 and a stable transformant expressing wild-type BRCA1 protein, termed UWB1.289+BRCA1; and b) wild-type chicken DT40 cells and lines with a BRCA1 or pol β gene deletion and a double knockout cell line for both BRCA1 and pol β genes. We note that the UWB1.289 and UWB1.289+BRCA1 cell lines used here were not used in a previous study of BER as a function of BRCA1 expression [9].

### Characterization of the BRCA1 cell lines

**Figure 1:**
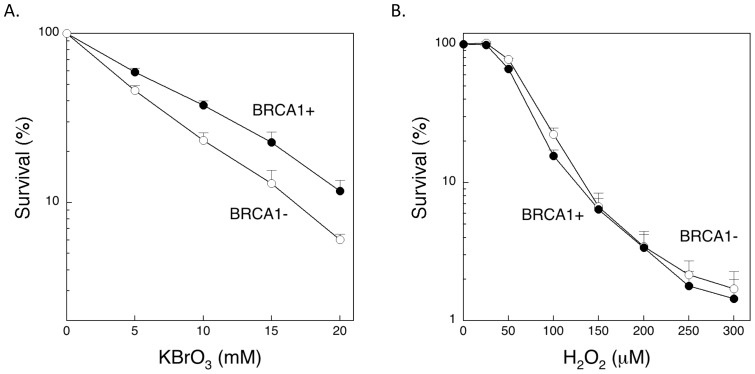
Characterization of sensitivity to oxidative DNA damage in human BRCA1 cell lines. Experiments were conducted as described under “Materials and Methods”. Human BRCA1 positive (+) and negative (−) cells were treated with A. KBrO_3_ or B. H_2_O_2_ for 1 h as indicated, and cell survival was measured by growth inhibition assay.

**Figure 2:**
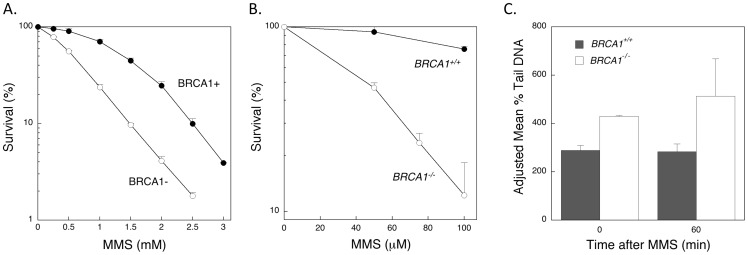
Characterization of MMS-induced DNA damage in human and DT40 BRCA1 cell lines. Experiments were conducted as described under “Materials and Methods”. A. Human BRCA1 positive (+) and negative (−) cells were treated for 1 h with MMS as indicated, and survival was measured. B. Chicken DT40 *BRCA1^+/+^* (wild-type) and *BRCA1^−/−^* cells were treated continuously with MMS, and survival was measured by clonogenic assay. Results with error bars represent the mean ± SE of at least 3 independent experiments; other data points represent the mean of two experiments. C. DT40 *BRCA1^+/+^* and *BRCA1^−/−^* cells were treated with 20 mM MMS for 20 min on ice and then subjected to the alkaline comet assay for measurement of strand breaks after 0 and 60 min repair. 100 cells were scored per slide, 2 slides per cell type and time, and migrated DNA was measured as the Mean % Tail DNA ± SD of 2 replicate slides. Mean % Tail DNA was normalized to the untreated Mean % Tail DNA in the respective cell line. The difference between cell types in level of DNA damage was significant (p = 0.003).

We initially examined cell survival of the BRCA1 cell lines after treatment with oxidative stress inducing agents (Figure 1). Cells were treated with the agents for 1 h, and then survival was measured after 6–7 days of culture. The BRCA1-deficient line was slightly more sensitive than the BRCA1 complemented cell line to potassium bromate (Figure 1A), but the two cell lines were equally sensitive to hydrogen peroxide (Figure 1B). These results from the hydrogen peroxide sensitivity experiment are different from those described by Alli et al. [9] using entirely different cell lines. In light of the resistance to oxidative stress-inducing agents, we examined the response to MMS-induced stress. Cells were treated with different levels of MMS for 1 h, and then survival was measured by growth inhibition assays (Figure 2A). The IC_50_ values from several experiments with the BRCA1 positive and negative cells were 1.4±0.1 and 0.6±0.05 mM, respectively. Results of MMS cytotoxicity assays with a DT40 cell line with a BRCA1 gene deletion and the isogenic wild-type control indicated that the *BRCA1^−/−^* cell line was hypersensitive to MMS (Figure 2B).

The comet assay (single cell gel electrophoresis) provides a means of detecting DNA damage (strand breaks, alkali-labile sites, cross-linking, adduct formation) in individual eukaryotic cells [31], [32], [33], The DT40 cell variants were used to confirm a DNA repair deficiency as a function of loss of BRCA1 expression. DNA strand breaks were measured by an alkaline comet assay in *BRCA1^+/+^* and *^−/−^* lines after treatment with 20 mM MMS for 20 min. After MMS treatment, cells were either analyzed immediately or cultured in fresh medium for 60 min without MMS to allow for repair before comet analysis. *BRCA1^−/−^* cells showed a higher level of strand breaks immediately after MMS treatment than *BRCA1^+/+^* (wild-type) cells (Figure 2C), and after repair for 60 min, similar results were obtained (Figure 2C). These results demonstrating more MMS-induced strand breaks in the *BRCA1^−/−^* cells than in wild-type cells were indicative of a defect in DNA repair as a function of loss of BRCA1 expression.

### Survival after MMS treatment in BRCA1- and pol β-deficient DT40 cells

**Figure 3:**
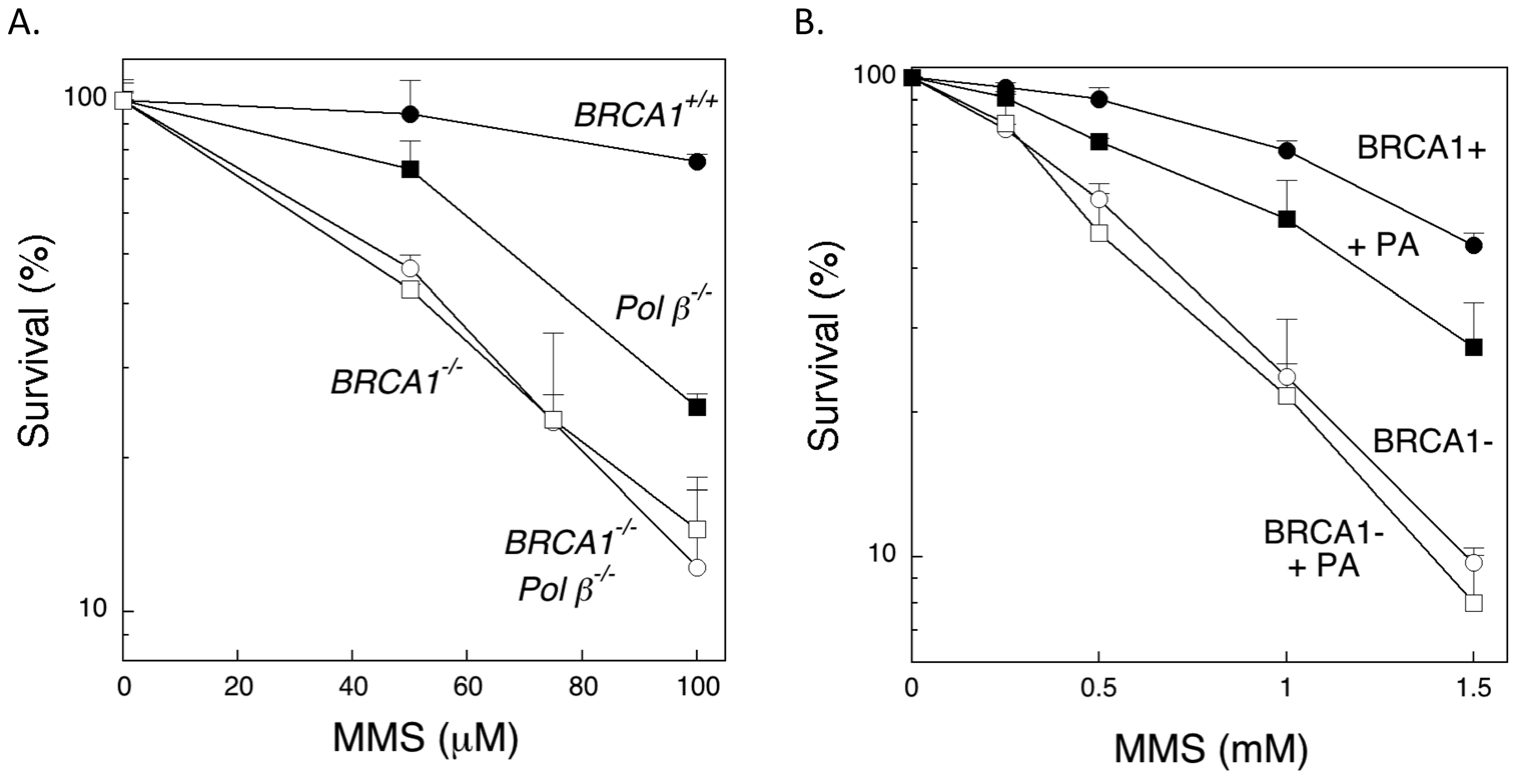
Analysis of DT40 and human cell survival after MMS treatment, as a function of BRCA1 and pol β expression and combined inactivation of BRCA1 and pol β. Experiments were conducted as described under “Materials and Methods”. A. DT40 *BRCA1^+/+^* (wild-type), *BRCA1^−/−^*, *Pol β^−/−^ and BRCA1^−/−^/Pol β^−/−^* double knockout cells were treated with MMS and survival was measured by colony formation. B. Human BRCA1-expressing (+) and negative (−) cells were treated with MMS in the presence or absence of the pol β inhibitor PA (300 µM for 24 h). Results with error bars represent the mean ± SEM of at least 3 independent experiments.

To examine a functional interaction between BRCA1 and pol β in DT40 cells, we measured MMS survival as a function gene deletion of BRCA1 and pol β. Wild-type (*BRCA1^+/+^*) and isogenic *BRCA1^−/−^*, *Pol β ^−/−^*, and *BRCA1^−/−^/Pol β ^−/−^* DT40 cell lines were exposed to a range concentrations of MMS. Both *BRCA1^−/−^* cells and *Pol β^−/−^* cells demonstrated MMS hypersensitivity (Figure 3A). Further, both *BRCA1^−/−^* and *BRCA1^−/−^/Pol β^−/−^* cells showed similar MMS sensitivity (Figure 3A). The lack of effect of pol β deletion in the BRCA1 negative background was striking. In the absence of pol β, deletion of BRCA1 produced additional sensitivity, i.e., beyond that observed with pol β deletion alone. These results suggested that BRCA1 and pol β could be involved in the same pathway toward protecting DT40 cells against cytotoxic effects of MMS treatment.

### Survival after MMS treatment in BRCA1-deficient and pol β-inhibited human cells

Cell survival analysis was conducted with the two human ovarian BRCA1 cell lines, but in this case pol β was inactivated using the inhibitor pamoic acid (PA) [34]. BRCA1-complemented and -deficient cells were pre-treated with PA before the usual 1 h MMS treatment, and then cells were incubated with growth medium containing PA for a total of 24 h. The BRCA1-complemented cells showed moderate MMS hypersensitivity with PA treatment (Figure 3B), consistent with blocking the BER activity of pol β [34]. The BRCA1-deficient cells were hypersensitive to MMS, but pol β inhibition by PA had no further effect, as if there was no pol β-mediated protective role in the absence of BRCA1 (Figure 3B). These results were consistent with those with DT40 cells, in that it appears BRCA1 and pol β may function in the same pathway toward protection against MMS-induced cytotoxicity, and the pol β protective role depended upon BRCA1 expression.

### Protein-protein interaction between human BRCA1 and pol β

**Figure 4:**
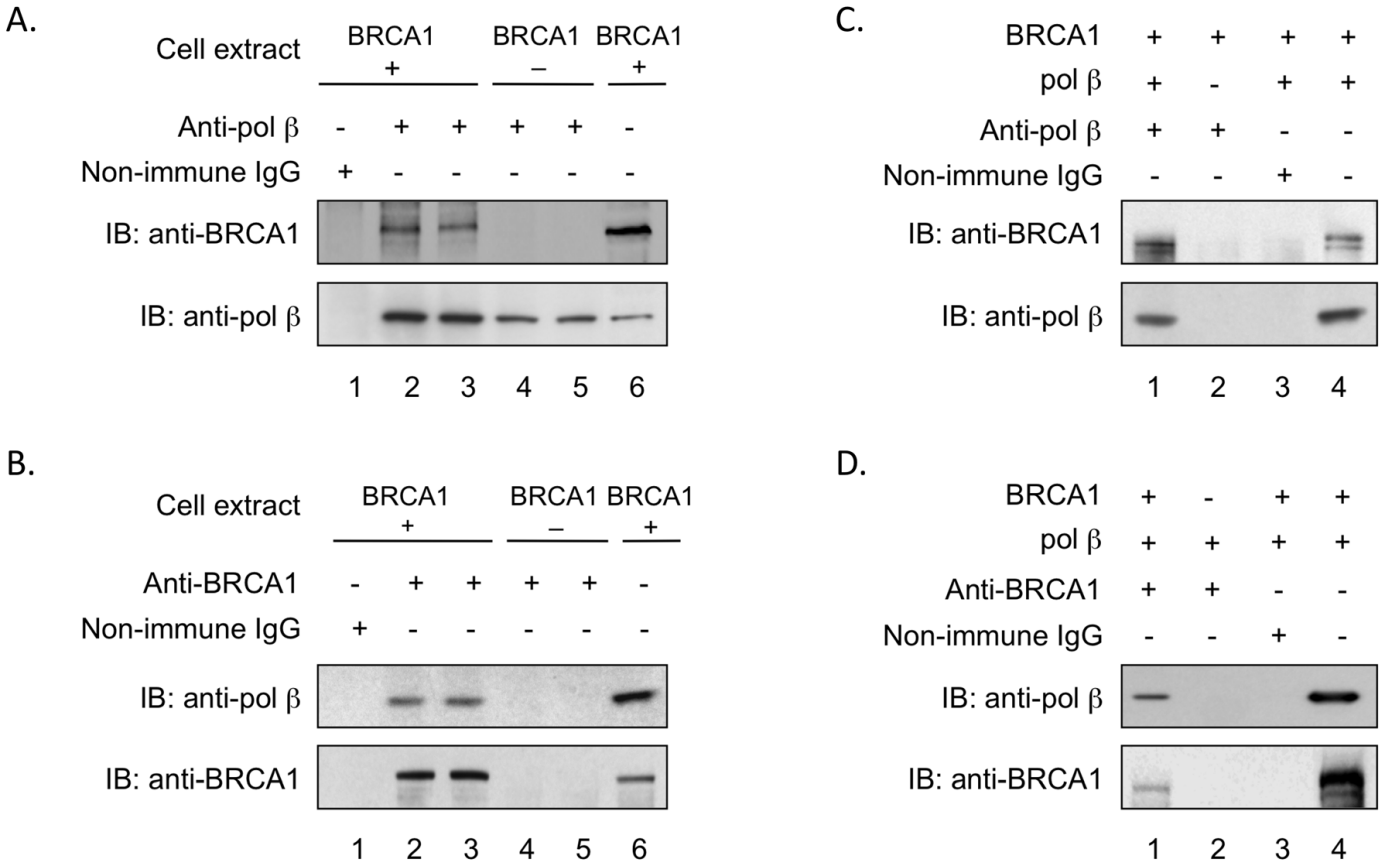
Co-immunoprecipitation analysis for human BRCA1 and pol β interaction. Experiments were conducted as described under “Materials and Methods”. Typical results obtained in at least three experiments are shown. Immunoprecipitated proteins were detected by SDS-PAGE and immunoblotting (IB). A. Cell extracts, as indicated, were immunoprecipitated with anti-pol β antibody (lanes 2–5) or non-immune IgG (lane 1). *Lanes* 2–3 and 4–5 are duplicates. Lane 6 corresponds to immunoblotting of the BRCA1-complemented extract, used here as a source of markers for BRCA1 and pol β. B, Cell extracts as indicated were immunoprecipitated with anti-BRCA1 antibody (lanes 2–5) or non-immune IgG (lane 1). Lanes 2–3 and 4–5 are duplicates, and lane 6 was as described in panel A. C. Purified human BRCA1 and pol β proteins were mixed and immunoprecipitated with anti-pol β antibody (lane 1) or non-immune IgG (lane 3). Pol β was omitted in lane 2, and lane 4 corresponds to the purified proteins used as markers. D. The same purified protein mixture as in panel C, except that immunoprecipitation was with anti-BRCA1 antibody (lane 1) or non-immune IgG (lane 3). BRCA1 was omitted in lane 2.

Since BRCA1 and pol β appeared to share a protective pathway against MMS-induced cytotoxicity, we looked for protein-protein interaction between these repair factors using co-immunoprecipitation analysis. Extracts were prepared from the human BRCA1-expressing and negative cells and subjected to immunoprecipitation using anti-pol β or anti-BRCA1 polyclonal antibodies. With the extract from BRCA1 positive (+) cells, BRCA1 was co-immunoprecipitated by the anti-pol β antibody, and with the extract from BRCA1 negative (−) cells there was no such immunoprecipitation, as expected (Figure 4A, compare lanes 2, 3 and 4, 5). A negative control immunoprecipitation with the BRCA1 positive cell extract using non-immune IgG was also negative, as expected (Figure 4A, lane 1). Immunoprecipitation of pol β in these experiments was confirmed by immunoblotting (Figure 4A, lanes 2–5), and the BRCA1-complemented extract served as a source of markers for BRCA1 and pol β in the blotting analysis (Figure 4A, lane 6). In reciprocal experiments, the anti-BRCA1 antibody immunoprecipitated pol β from the BRCA1 positive cell extract, but not from the BRCA1 negative cell extract, as expected (Figure 4B, lanes 2–5). A control immunoprecipitation with non-immune IgG was also negative, as expected (Figure 4B, lane 1), and immunoblotting confirmed the immunoprecipitation of BRCA1 (Figure 4B, lanes 2 and 3).

Next, we examined the possibility of direct protein-protein interaction between purified human BRCA1 and pol β proteins. Consistent with the result with cell extracts, the anti-pol β antibody immunoprecipitated BRCA1 (Figure 4C, lane 1), and the immunoprecipitates with BRCA1 protein alone or non-immune IgG were negative for BRCA1 (Figure 4C, lanes 2 and 3). The anti-BRCA1 antibody precipitated pol β from the protein mixture (Figure 4D, lane 1), but no such immunoprecipitation was observed with non-immune IgG or with pol β alone (Figure 4D, lanes 2 and 3). These results suggest that human BRCA1 and pol β are capable of direct protein-protein interaction.

### Analysis of BER capacity

Based on the results described above, we determined BER capacity as a function of BRCA1 expression in the human cell lines. Initially, an *in vivo* plasmid-based assay that relies on luciferase expression as a function of repair of uracil-DNA was used. This assay is considered as a reliable method for examining monofunctional DNA glycosylase-initiated and pol β-mediated BER *in vivo*. The assay is designed so that uracil is placed at a stop codon within the luciferase gene, and luciferase protein is expressed only if the uracil is repaired [21]. Both of the BRCA1 cell lines were transfected with AM1-U and the positive control plasmid along with pGL4.75, and luciferase activity was measured continuously during 18 h of culture, as described previously (Figure S2A). The repair activity of the BRCA1 negative and positive cells was found to be similar. Thus, these results from an *in vivo* BER assay failed to reveal a requirement of BRCA1 for repair of the uracil lesion.

To further evaluate BER in the BRCA1-expressing and -deficient cells, we conducted *in vitro* BER assays, again using uracil-DNA as substrate. First, the expression levels of several BER factors were confirmed by immunoblotting analysis of the extracts (Figure S2B). The analysis confirmed the presence in both cell lines of generally similar levels of DNA repair proteins. The expression levels of PARP-1, ligase I and ligase III were slightly higher in BRCA1 negative than in BRCA1 positive cells, but pol β, FEN1 and XRCC1 levels were similar.

Full-length BRCA1 protein was expressed only in the BRCA1 positive (+) cells (Figure S2B), but we noted the truncated form of BRCA1 was expressed in both cell lines, as expected, since the BRCA1 functionally negative cells have a mutation in exon 11 of the BRCA1 gene leading to a truncated form of the protein [35].

Next, the extracts were subjected to the *in vitro* BER assay involving an oligonucleotide substrate with a uracil lesion. This duplex 34-mer substrate, with uracil at position 15, was incubated with the extracts from BRCA1 cells along with ^32^P-labeled nucleotide substrate. After the incubations, analysis revealed that gap-filling intermediates and ligated BER products had been formed depending on the incubation time (Figure S2C). The amounts of these products were similar with the extracts from both BRCA1 cell lines. These results were consistent with the results from the *in vivo* BER assay described above, and failed to reveal a requirement for BRCA1 in monofunctional DNA glycosylase-initiated BER.

### Immunofluorescence imaging of BRCA1 and pol β in human cells

Despite obtaining negative data in the BER assays, we further examined the relationship between BRCA1 and pol β in protection against DNA damage, as suggested by the MMS survival results in Figure 2. Immunofluorescence imaging experiments were conducted with both of the human BRCA1 cell lines. In these experiments, we applied an alternate approach of inducing DNA damage and followed recruitment of BRCA1 and pol β proteins. Laser-delivered microirradiation, producing oxidative DNA base damage and strand breaks was used, along with antibodies to BRCA1 and pol β. If BRCA1 was indeed involved in BER of this damage, it would be expected to exhibit rapid recruitment to the sites of irradiation, i.e., within the first 60 s as is known to be the case for pol β [36].

**Figure 5:**
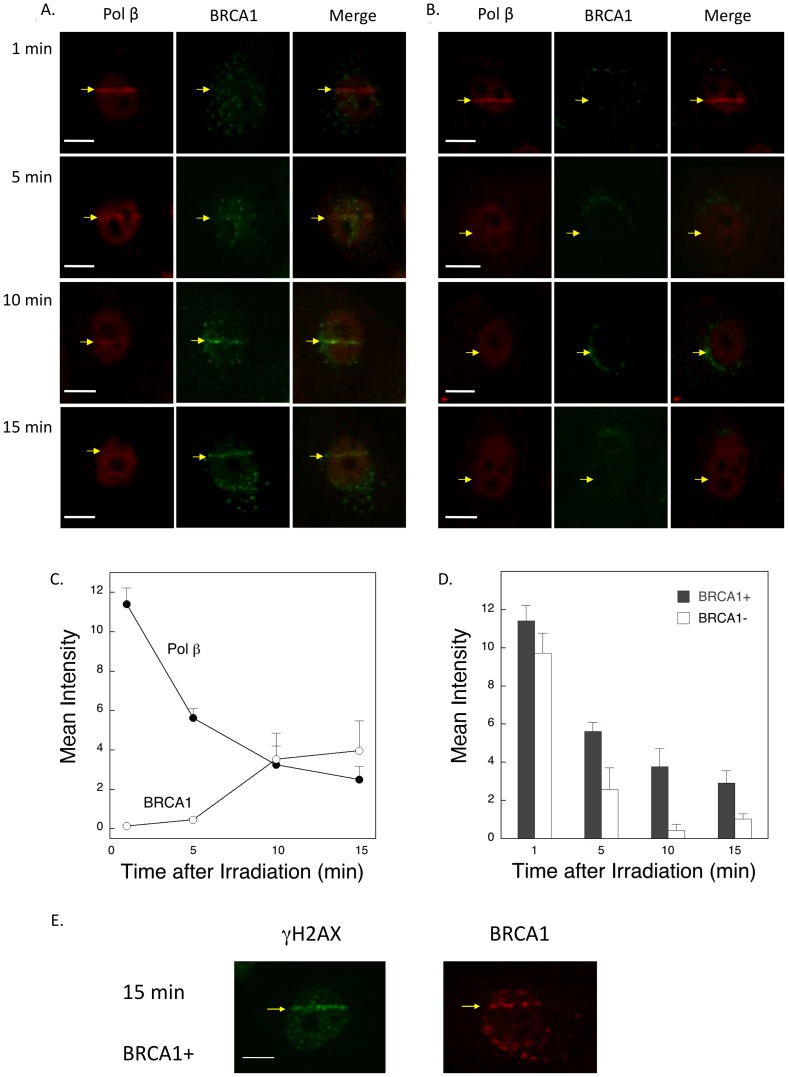
Immunofluorescence imaging of pol β and BRCA1 in human cells using anti-pol β and anti-BRCA1 antibodies. Experiments were conducted as described under “Materials and Methods,” and typical results are shown. A. BRCA1 positive and B. BRCA1 negative cells were irradiated in stripes and then allowed to repair for the indicated times before assessment for pol β and BRCA1 recruitment, as indicated. C. Summary of intensities of the pol β and BRCA1 signals as a function of time after irradiation of the BRCA1-expressing cells. Quantification was of at least 18 cells for each protein with error bars representing mean ± SD. D. Summary of intensities of the pol β signals, as a function of time after irradiation, in the BRCA1 positive cells (+, solid bars) and negative cells (−, open bars). Values plotted represent mean ± SD corresponding to 4 cells at each point. E. Imaging of γ-H2AX in the BRCA1-expressing (+) cells 15 min after irradiation. Bars represent 10 µm.

With the BRCA1 complemented cells (Figure 5A), there was strong pol β recruitment within 60 s after irradiation, whereas BRCA1 recruitment was not detected at all at this time point. The pol β signal declined after 1 min and was much lower by 15 min after irradiation. In contrast, BRCA1 recruitment was first weakly visible at 5 min and recruitment was strong at 15 min. The patterns of recruitment and decay of the signal for BRCA1 and pol β were quite different (Figure 5C), and the pattern with BRCA1 did not appear to be consistent with a role in BER, since BER factors are known to be recruited in an early phase (<1 min) after irradiation. The recruitment pattern of BRCA1 did, however, seem consistent with its known role in DSB repair [37], and in experiments not shown, the pattern of γ-H2AX recruitment was similar to that for BRCA1. As expected, recruitment of BRCA1 was not observed with the BRCA1 negative cells.

Next, we examined pol β recruitment in the BRCA1 negative cells (Figure 5B). Strong recruitment again was observed at 60 s after irradiation. However, the decay in the pol β signal was faster than that observed with the BRCA1 complemented cells, and was negligible at 10 and 15 min (Figure 5B). This pattern for pol β was verified when the results were quantified (Figure 5D). Finally, in the BRCA1 positive cells at 15 min after irradiation, γ-H2AX and BRCA1 co-localized at the stripe of laser irradiation (Figure 5E). Overall, these results with laser irradiation and immunofluorescence imaging indicated that the presence of BRCA1 at focal stripes of DNA damage was required for prolonged retention of pol β, most likely reflecting ongoing DSB repair.

**Figure 6:**
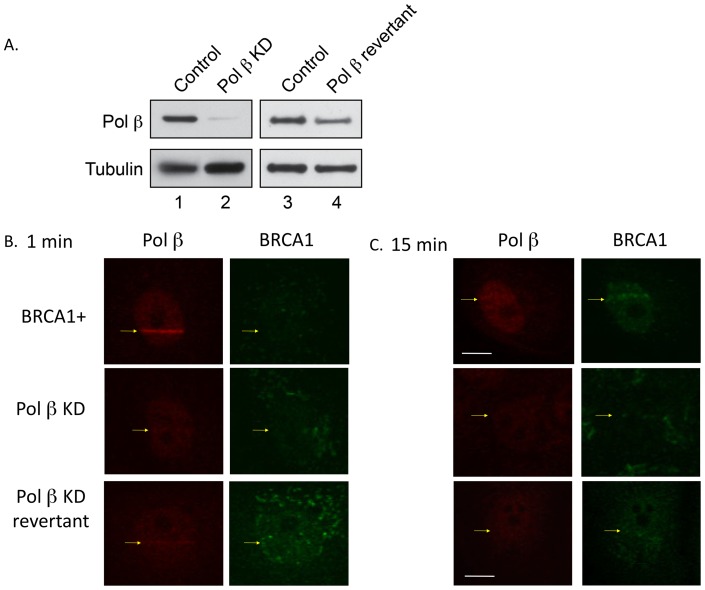
Analysis of pol β KD cells. A. Western blotting analysis of pol β in a pol β knockdown (KD) cell line and in a partial revertant. B and C. Immunofluorescence imaging of the recruitment of pol β and BRCA1 at 1 and 15 min after irradiation of BRCA1+, pol β KD and pol β KD revertant cell lines. Bars represent 10 µm.

A reciprocal experiment evaluated the possibility that pol β might be required for BRCA1 recruitment at the focal stripes of DNA damage. For this experiment, a cell line with knockdown (KD) of pol β was developed in the human BRCA1-expressing cells described above. Immunoblotting of extract from the pol β KD cells confirmed the strong reduction (>90%) of pol β (Figure 6A, lanes 1 and 2). Another cell line was studied where the level of pol β had partially reverted to a level approximately 30% of the wild-type level (Figure 6A, lanes 3 and 4). These two cell lines were used in imaging experiments similar to those described above. As expected, at 1 min after irradiation, pol β recruitment was observed in the BRCA1 positive (+) cells, but not in the pol β KD cells, and a very minor signal of pol β recruitment was observed in the pol β KD revertant cells (Figure 6B). BRCA1 recruitment was not observed at this early time after irradiation, again as expected. At 15 min after irradiation, pol β and BRCA1 recruitment were observed as usual in the BRCA1 positive (+) cells (Figure 6C). In contrast, BRCA1 recruitment was not observed in the pol β KD cells (Figure 6C). Thus, pol β expression appeared to be required for BRCA1 recruitment. In line with this, a weak BRCA1 recruitment was seen with the pol β revertant cells (Figure 6C) and a faint band of pol β recruitment could be seen at 15 min after irradiation.

## Discussion

It is known that BRCA1 participates in DSB repair [6], [38] and in BER of 8-oxoguanine in human and mouse mammary epithelial cells [9]. In the present study we explored the possibility that BRCA1 might also be involved, along with pol β, in BER repair of alkylation base damage. Cellular phenotypes reflecting BER status were evaluated as a function of BRCA1 expression. These included MMS hypersensitivity (Figure 2) and analysis of combined pol β/BRCA1 function in protection against MMS-induced cytotoxicity (Figure 3). In comparing the BRCA1 cell lines, the MMS sensivity results were consistent with a role of BRCA1 in BER. Thus, the BRCA1 negative cells were hypersensitive to MMS, and more specifically, in the BRCA1 negative cell background, the MMS-sensitivity phenotype of pol β-dependent BER was completely absent. However, we consider these results indicating MMS treatment phenotypes as not being strictly diagnostic of a BER deficiency, but instead could reflect the status of multiple other protective systems in the cells [13]. Therefore, we evaluated the BER capacity of the human ovarian BRCA1 cell lines more directly using a plasmid-based *in vivo* BER assay and an *in vitro* BER assay with cell extracts. In both of these assays, there were no differences in BER as a function of BRCA1 expression. We concluded from these experiments that BRCA1 was not required for the monofunctional glycosylase-initiated BER activities measured in the *in vivo* BER assay and *in vitro* BER assay with uracil-DNA. These results are in contrast to the results obtained by Alli et al. using an *in vivo* host-cell reactivation assay with oxidized DNA [9] and human breast cell lines.

Two surprising findings emerged from the present study. First, pol β and BRCA1 appeared to have a functional interaction, observed in both DT40 and human cell lines, in protecting cells against MMS-induced cytotoxicity (Figure 3); yet, as noted above, pol β had no protective effect in the absence of BRCA1. Further, in light of the lack of a BRCA1 role in the direct BER assays used here, it is possible that the pol β and BRCA1 relationship reflects a role of pol β in DSB repair. Second, pol β and BRCA1 were capable of protein-protein interaction in co-immunoprecipitation experiments. A partnership between pol β and BRCA1 has not been observed previously and a role of pol β in DSB repair also has not been reported.

The results of immunofluorescence imaging experiments with the human cell lines and laser-delivered DNA damage did not support a role for BRCA1 in BER (Figure 5). The BRCA1 protein was not detected at focal sites of DNA damage on the rapid time scale expected for a BER factor, such as pol β or XRCC1. Instead, the imaging results at longer times after irradiation indicated a co-localization between BRCA1 and pol β at focal sites of damage that were also positive for γ-H2AX recruitment. The amount of pol β localization at these sites corresponded to persistence of a fraction of the pol β protein recruited at 1 min after irradiation. Proteins recruited at damage sites at 15 min after irradiation are more likely involved in DSB repair. The observations that pol β recruitment at 15 min after irradiation was dependent on BRCA1 expression and conversely that recruitment of BRCA1 was dependent on pol β expression are consistent with a functional partnership between these proteins in DSB repair. The molecular mechanism of the repair partnership between pol β and BRCA1 remains to be determined.

Finally, in light of the results described here, it is interesting to consider the possibility that the MMS sensitivity (shown in Figure 3) and the micro-irradiation imaging results (shown in Figures 5 and 6) point to the same phenomenon, a joint role of BRCA1 and pol β in DSB repair. First, as noted already, the complete absence of a pol β hypersensitivity phenotype in the *BRCA1^−/−^* background was striking. Thus, the protective effect of pol β strictly depended on the presence BRCA1. Since we expect the MMS protective effect of pol β to be due to its dRP lyase activity, these present results raise the possibility that pol β could fulfill an end-tailoring role in DSB repair. A role of this sort has been proposed already for the Ku protein complex in non-homologous end joining [39]. Finally, the MMS sensitivity experiments and micro-irradiation imaging experiments both involve strand break intermediates of BER. In the case of MMS treatment this is triggered by alkylation and oxidation DNA base damage and in the case of micro-irradiation by oxidative damage and direct strand breaks. More experiments will be necessary to explore these interesting possibilities.

## Supporting Information

Figure S1 Genomic DNA characterization of the pol β gene in the pol β and BRCA1 double knockout cell line produced by disruption of the pol β gene in *BRCA1^−/−^* DT40 cells. A. Southern blot analysis of the pol β gene disruptions (*−*/*−*). Hind III-digested genomic DNA was used to confirm the targeted disruption of the pol β locus using the probe shown in the physical map. Analysis of the intact pol β gene in the BRCA1 knockout (−/−) cell line used in pol β disruption is shown in lane 1. Confirmation of pol β gene disruption in a cell line with the intact BRCA1 gene is shown in lane 3. Confirmation of pol β gene disruption in the cell line with the disrupted BRCA1 gene is shown in lane 2. B. Physical maps representing the chicken pol β locus and targeted deletion locus. A solid box represents the exons and H represents the Hind III sites. ‘Probe’ represents the location of the DNA used in the Southern blot analysis in A.
(TIFF)

Figure S2 BER in human cell lines. A. Assay for uracil-DNA BER *in vivo* using a plasmid-based assay in the two human cell lines. Experiments were conducted as described under “Materials and Methods”. Cell lines were transfected with the BER reporter plasmid and luciferase activity was measured in BRCA1 positive (+) and negative (−) cells after 18 h of repair. Relative repair-dependent luciferase activity is shown. Results are the average of 4 experiments ± SE. B. Western blotting analysis to estimate levels of BRCA1 and six known BER proteins in the BRCA1 positive (+; lane 1) and negative (−, lane 2) cell lines. GAPDH was used as loading control (bottom panel). C. Assessment of uracil-DNA BER *in vitro* using extracts from human BRCA1 positive (+; lanes 1–3) and negative (−; lanes 4–6) cells. Experiments following incorporation of ^32^P-labeled dNTP into the oligonucleotide substrate DNA were conducted as described under “Materials and Methods” and a typical result of 3 individual experiments is shown. Incubation times are as indicated above the gel. Intermediate BER products (1-nt gap-filling) and ligated BER products are indicated.
(TIFF)
