# Supplementary figures and images for: Interaction between DNA Polymerase β and BRCA1
Source: PLoS One. 2013 Jun 27;8(6):e66801. doi: 10.1371/journal.pone.0066801 (PMC3694962; doi:10.1371/journal.pone.0066801)

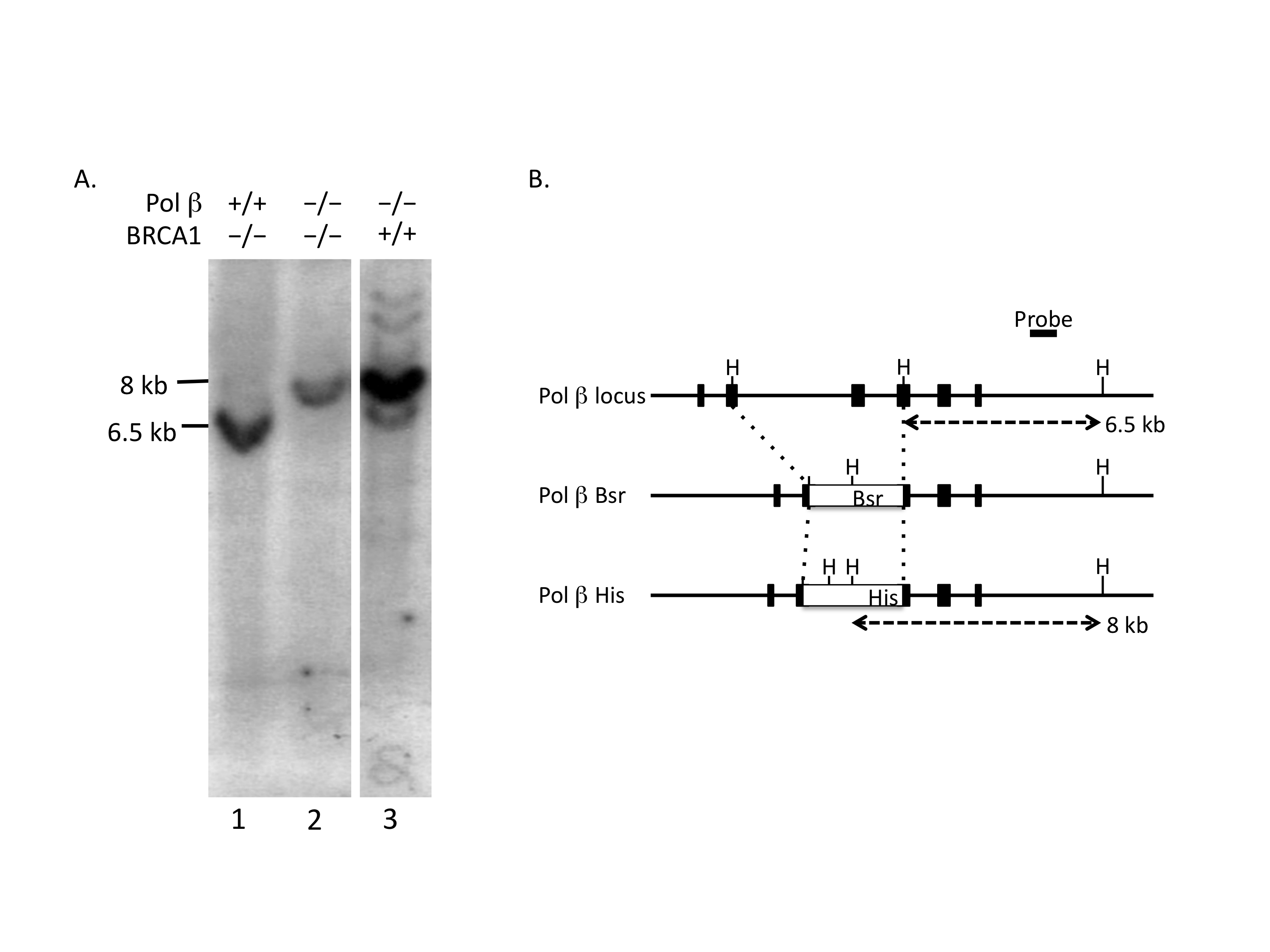

Supplement: Figure S1 — Genomic DNA characterization of the pol β gene in the pol β and BRCA1 double knockout cell line produced by disruption of the pol β gene in BRCA1−/− DT40 cells. A. Southern blot analysis of the pol β gene disruptions (−/−). Hind III-digested genomic DNA was used to confirm the targeted disruption of the pol β locus using the probe shown in the physical map. Analysis of the intact pol β gene in the BRCA1 knockout (−/−) cell line used in pol β disruption is shown in lane 1. Confirmation of pol β gene disruption in a cell line with the intact BRCA1 gene is shown in lane 3. Confirmation of pol β gene disruption in the cell line with the disrupted BRCA1 gene is shown in lane 2. B. Physical maps representing the chicken pol β locus and targeted deletion locus. A solid box represents the exons and H represents the Hind III sites. ‘Probe’ represents the location of the DNA used in the Southern blot analysis in A. (TIFF) [file pone.0066801.s001.tiff]

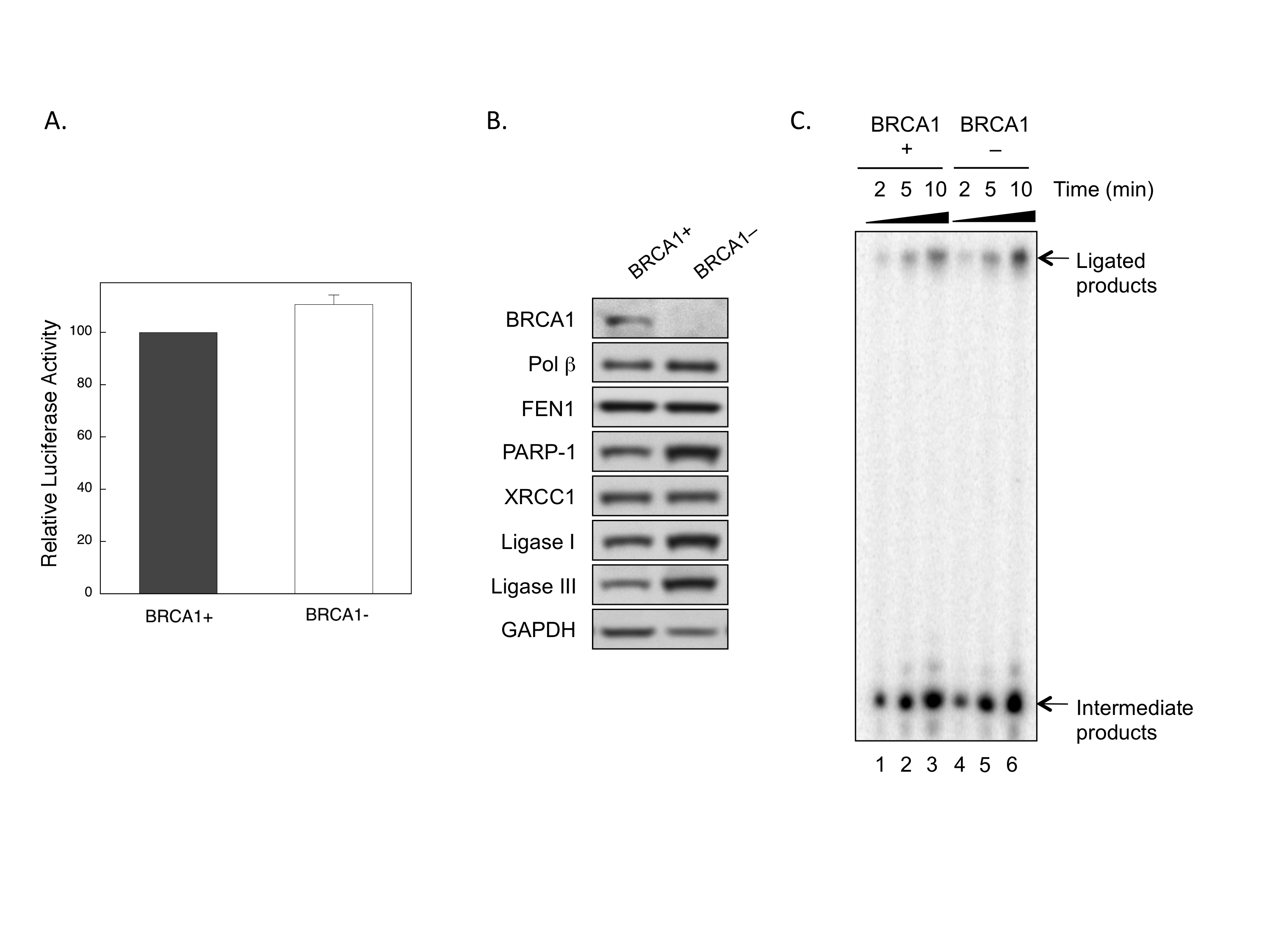

Supplement: Figure S2 — BER in human cell lines. A. Assay for uracil-DNA BER in vivo using a plasmid-based assay in the two human cell lines. Experiments were conducted as described under “Materials and Methods”. Cell lines were transfected with the BER reporter plasmid and luciferase activity was measured in BRCA1 positive (+) and negative (−) cells after 18 h of repair. Relative repair-dependent luciferase activity is shown. Results are the average of 4 experiments ± SE. B. Western blotting analysis to estimate levels of BRCA1 and six known BER proteins in the BRCA1 positive (+; lane 1) and negative (−, lane 2) cell lines. GAPDH was used as loading control (bottom panel). C. Assessment of uracil-DNA BER in vitro using extracts from human BRCA1 positive (+; lanes 1–3) and negative (−; lanes 4–6) cells. Experiments following incorporation of 32P-labeled dNTP into the oligonucleotide substrate DNA were conducted as described under “Materials and Methods” and a typical result of 3 individual experiments is shown. Incubation times are as indicated above the gel. Intermediate BER products (1-nt gap-filling) and ligated BER products are indicated. (TIFF) [file pone.0066801.s002.tiff]
